# Novel Viral Vectors for Gene Therapy

**DOI:** 10.3390/v16030387

**Published:** 2024-03-01

**Authors:** Ottmar Herchenröder, Brigitte M. Pützer

**Affiliations:** Institute of Experimental Gene Therapy and Cancer Research, Rostock University Medical Center, 18057 Rostock, Germany

Viral vectors are gene transfer tools assembled from the backbones of naturally occurring viruses. By definition, these vehicles that transfer nucleic acids are replication-incompetent but deliver assigned payloads into eukaryotic cells by a process called transduction. For decades, numerous viral vector systems that influence cells or tissues have been used to perform basic and preclinical research. Over time, some virus-derived vectors found their way into clinical practice.

When the severe acute respiratory syndrome coronavirus type 2 (SARS-CoV-2) began to quickly spread at the turn of 2019/2020, ultimately leading to a pandemic spanning the globe and paralyzing the world, vaccines had to be developed quickly. Besides the classical methodologies for vaccine production such as inactivated viruses or preparations based on viral proteins either harvested or produced in recombinant settings, two rather novel techniques gained the upper hand. In less than twelve months, nucleic acid-based as well as adenovirus-derived vector systems were approved as vaccines to combat COVID-19. Both types of vaccines transmit a genetic blueprint into muscle tissue, enabling the organism to build the spike protein of SARS-CoV-2 and prime the immune system against the disease. Early on in 2021, both biotechnological achievements helped to control the spread of SARS-CoV-2 and to reduce the severity of the disease, thereby reducing the burden on healthcare systems. In addition, they allowed societies to return to a sense of normalcy. 

On the other hand, the use of viral vectors as a vaccination tool during the COVID-19 crisis has taught us the lesson that there are still some issues to be solved to avoid unwanted serious side effects and make this next-generation gene delivery technology, proven successful in many approaches, clinically usable. The development of novel viral vectors that meet the requirements of future patients warrants their individual optimization and adaptation for different applications in gene therapy, cancer treatment, vaccine development, and cell reprogramming [1]. Since its approval as the first gene therapy product, a plethora of strategies using adenovectors emerged, including conditionally replicative oncolytic viruses [2], less-immunogenic, genetically stable high-capacity adenovirus-derived vehicles, which allow long-term episomal persistence of transgenes in non-dividing cells [3], or customized adenovectors for targeted transduction in vivo. Viral vectors are now widely used and the number of approved therapies is only expected to increase. To keep up with the increasing demand, the challenges and strategies for faster patient access need to be addressed. The editors believe that this Special Issue provides some optimistic answers.

In three articles on adenoviral vectors, we learn about the challenges in setting up robust platforms for vector production. Researchers from Witten in Germany and Bordeaux in France show that the construction and fabrication of a DNA-virus-based vector to transfer genetic information from an RNA virus are not trivial. Scientists residing in Montreal in Canada present improved methodologies to manufacture vectors that do not contaminate replication-competent entities. A team from Cardiff, UK, presents data on improving cell specificity and selectivity of adenoviral vector particles to ultimately improve oncolytic viruses. 

Two papers deal with adeno-associated-virus (AAV)-derived gene ferries. The group led by Sarah Wootton from the University of Guelph, Canada, improved AAV by pseudotyping the particles with animal virus-derived capsid sequences. Victor Weiss and colleagues in Vienna, Austria, provide a new technique using a sophisticated apparatus to characterize AAVs.

A review by Wang and Shao from Tianjin, China, looks at innate immune responses to viral vectors and on how to prevent these responses. A consortium from Greece and Cyprus explains how the researchers optimized a lentiviral vector built to threat sickle cell disease. 

In two back-to-back articles, both authored by Randal N. Johnston from Calgery, Canada, the researchers from Canada and Japan describe the properties of oncolytic reoviruses. These tools show anti-cancer activity in conjunction with chemotherapy. Further activity is unveiled as these oncolytic viruses can also infect and destroy pluripotent stem cells. 

An illustrious panel of Central European senior scientists discusses the need for disinfectants in the context of modified viruses and vectors.

To round up this Special Issue, Kenneth Lundstrom from Switzerland presents a seminal review on the prospects of the current frontline vector systems. In his comprehensive article, the author covers most aspects of the vectors currently in use, including oncolytic viruses. 

As we look forward, it is essential to continue researching viral vectors including cutting-edge technologies such as artificial intelligence for the production of targeted and more efficient gene ferries, ensuring transparency in reporting adverse events, and making data-driven decisions to balance risks and benefits. The incidents related to these vector-derived vaccines should not deter us from recognizing the immense value of viral vectors in medical applications. We believe that every author of this Special Issue would agree with this statement.
